# Akt1 Enhances CA916798 Expression through mTOR Pathway

**DOI:** 10.1371/journal.pone.0062327

**Published:** 2013-05-08

**Authors:** Yu-Liang Wang, Bing-Jing Zhu, Zhan-Zhong Qi, Hai-Jing Wang, Xiang-Dong Zhou

**Affiliations:** Department of Respiratory Medicine, Southwest Hospital, The Third Military Medical University, Chongqing, China; H.Lee Moffitt Cancer Center & Research Institute, United States of America

## Abstract

Multi-drug resistance leads to the failure of chemotherapy for cancers. Our previous study showed that overexpression of CA916798 led to multi-drug resistance. However, the underlying mechanisms remain unknown. In the current study, we observed that the levels of phosphorylated AKT, phosphorylated mTOR and CA916798 all increased in the drug resistant human adenocarcinoma samples and paralleled with the change of drug resistance. The results of immunofluorescence and Co-IP indicated that the positive correlation of CA916798 expression with AKT1 activation might be associated with drug resistance of lung adenocarcinoma. Furthermore, AKT1 stimulated CA916798 expression through mTOR pathway in both A549 and A549/CDDP cell lines, which was also observed in the xenografted tumor in nude mice. The results showed that CA916798 located in the downstream of PI3K/AKT/mTOR pathway. Inhibition of PI3K by LY294002 could efficiently reduce CA916798 expression and tumor size in vivo as well. Additionally, LY294002 combined with rapamycin inhibited CA916798 expression and tumor size stronger than LY294002 alone. Our findings may also provide a new explanation for synergistic anti-tumor effects of PI3K and mTORC1 inhibitors.

## Introduction

Lung cancer is the primary death cause for human beings in cancers [1]. Chemotherapy is one of effective methods to treat lung cancer. However, some lung cancer cells develop resistance to chemotherapeutics including cisplatin, carboplatin, gemcitabine, vincristine, and pacilitaxel, which makes lung cancer much more difficult to cure [2], [3], [4], [5]. A better understanding of mechanisms of multi-drug resistance is undoubtedly necessary and will be beneficial for clinicians to design more effective therapy.

CA916798 is a novel gene found by using suppression subtractive hybridization from SPCA-1/CDDP, a human adenocarcinoma multi-drug resistance cell line [6]. Further studies indicate that CA916798 is a drug resistance-related gene. Higher expression of CA916798 could be detected in A549/CDDP cells, a multi-drug resistance cell line, as compare with its parental A549 cells. And over expression of CA916798 led to multidrug resistance in H446 cell [7], [8], while inhibition of CA916798 reversed the drug resistance capability of multi-drug resistance cell line A549/CDDP [8]. However, the mechanisms of CA916798 underlying multi-drug resistance are still unknown.

PI3K/AKT pathway is essential for multi-drug resistance, and inhibition of this signaling pathway can reverse drug resistance of tumors to chemotherapies so that treatment becomes more effectively [9], [10]. Many multi-drug related signaling pathways are correlated with PI3K/AKT pathway, such as survivin, caspases and p53 [11], [12]. We found that AKT1 phosphorylation was correlated with CA916798 expression in a pulmonary adenocarcinoma cell line, which was even stronger in A549/CDDP cell line than in normal A549 cell line. Thus we hypothesize that correlation of CA918798 with PI3K/AKT pathway may lead to multi-drug resistance. Herein, we examined the relationship between PI3K/AKT pathway and CA916798, and explored the mechanisms by which CA916798 led to resistance of chemotherapy.

## Materials and Method

### Ethics Statement

The nude mice experiment in this study was carried out in strict accordance with the recommendations in the Guide for the Care and Use of Laboratory Animals of the National Institutes of Health. The protocol was approved by the institutional animal care and use committee of the third military medical university. All surgery was performed under chloral hydrate anesthesia, and all efforts were made to minimize suffering.

The human lung cancer specimens study was approved by ethics committee of the First affiliated hospital of the Third Military Medical University. Each patients in this study has given written informed consent (as outlined in PLOS consent form) to publish these case details.

### Cell Culture

Human adenocarcinoma A549 cell line (which was purchased from the Cell Bank of the Chinese Academy of Sciences, Shanghai, China) was cultured in RPMI1640 containing 10% heat-inactivated fetal bovine serum, 100 units/mL penicillin G, and 100 mg/mL streptomycin in a 37°C and 5% CO2 incubator. Multi-drug resistant cells (A549/CDDP) were established by stepwise increasing cisplatin (CDDP) concentrations (Table S1), until the cells can generate normally in culture media containing 2 µg/mL CDDP. Usually A549/CDDP was cultured in the medium containing 2 µg/mL CDDP, and then the cells were cultured without CDDP for 1 month before the analysis was done.

### Plasmid Transfection

A549 or A549/CDDP cells were plated at 2×10^5^cells/well in six-well plates, cultured overnight, and transfected with 2 µg plasmid DNA by using polyjet reagent (Signal Gene Laboratories) according to the manufacturer’s instruction. After transfection, the cells were passaged in the medium containing G418 to ensure that the cells transfected with plasmids successfully could form cell clones, and then the stable transfected cells were selected by amplification culture. The plasmids are plncx-AKT1 and plncx-AKT1-K179M (Addgene, USA) [13], and pgcsi-CA916798–ShRNA [8] as well as corresponding empty vectors.

For AKT1 siRNA and Rictor siRNA transfection, A549 or A549/CDDP cells were plated at 0.5×10^5^cells/well in 24 well plates, cultured overnight, and transfected with 1 µg DNA by using Lipofectamin™ 2000 reagent (Invitrogen, USA) according to the manufacturer’s instruction. Cell was collected for corresponding analysis after 24 hours transfection. AKT1 siRNA and control siRNA were purchased from Santa Cruz Biotechnology (Santa Cruz, CA, USA), while Rictor siRNA was purchased from Cell Signaling Technology (MA, USA).

### Real-time Reverse Transcription PCR (RT-PCR)

Total RNA was extracted from the cells by using TRIzon Reagent (Cwbiotech, China) according to the manufacturer’s instruction. One microgram of total RNA was transcribed to cDNA by MMLV reverse transcriptase cDNA kit (Cwbiotech, China). RT-PCR analysis was done using transcribed cDNAs as the template by Sybgreen PCR kit (Cwbiotech, china). Levels of GAPDH were used as the internal control. Primers for the PCR analysis were listed as following:

AKT1: Sense primer, TCCTCCTCAAGAATGATGGCA; Anti-sense primer, GTGCGTTCGATGACAGTGGT. mTOR: Sense primer, TCCGAGAGATGAGTCAAGAGG; Anti-sense primer, CACCTTCCACTCCTATGAGGC. GAPDH: Sense primer, AAGGTGAAGGTCGGAGTCAAC; Anti-sense primer, GGGGTCATTGATGGCAACAATA; CA916798: Sense primer, GCTTCCTCCTCAACCTCGTCCT; Anti-sense primer, GTAGCCCACTTATCCACCTTCTCC. Rictor: Sense primer, GCTAGGTGCATTGACATACAACA; Anti-sense primer, AGTGCTAGTTCACAGATAATGGC.

### Western Blotting

Cells were washed with cold PBS, and then lysed in ice-cold cell lysis buffer [20 mM Tris PH7.5, 150 mM NaCl, 1% TritonX-100, 2.5 mM sodium pyrophosphate, 1 mM EDTA, 1%Na_3_VO_4_, 0.5 µg/ml leupeptin, 1 mM phenylmethanesulfonylfluoride (PMSF)]. The lysates were collected by scraping from the plates, and then centrifuged at 14,000×g for 10 min at 4°C. Total protein concentrations in the supernatant were determined by Bicinchoninic Acid assay kit (Cwbiotech, china). The proteins were resolved on 10% SDS–PAGE, transferred onto 0.22 µm PVDF membrane, and incubated with antibodies against CA916798, Akt1, phospho-Akt1 (Ser473), P70S6K, phosphor-P70S6K (serThr421/Ser424) and GAPDH, respectively. For Rictor, mTOR and phospho-mTOR (Ser2448), the proteins were resolved on 6% SDS-PAGE, and transferred to 0.45 µm PVDF membranes. Antibodies against Akt1, mTOR protein, phospho-mTOR (Ser2448), P70S6K protein, phosphor-P70S6K (Thr421/Ser424), and Rictor were purchased from Cell Signaling Technology. GAPDH antibody was purchased from Beyond Time Biotechnology. Phospho-Akt1 antibody was purchased from Santa Cruz Biotechnology, and rabbit anti human polyclone CA916798 antibody was made and kindly offered by Dr. Haijing Wang [8]. The membranes were washed with TBS buffer containing 0.05% Tween 20, and then incubated with the corresponding HRP-linked secondary antibodies. The levels of specific proteins were detected with Enhanced Chemiluminescence reagent (Cwbiotech, China).

### Immunofluorescence

A549 or A549/CDDP cells were cultured on glass slides. Cells were fixed and then stained with antibodies against phospho-Akt1 (Ser473) and CA916798 respectively. The corresponding FITC or Cy3-conjugated secondary antibodies (Cwbiotech, China) were used as secondary antibodies. Images were acquired by laser confocal, and the expression level of target gene was analysis by IPP5.0 Software.

### Co-Immunoprecipitation (Co-IP)

Cells were seeded in 75 cm^2^ plates and properly treated. Collected cell were lysed in ice-cold cell lysis buffer containing 20 mM Tris, 150 mM NaCl, 1% TritonX-100, 2.5 mM sodium pyrophosphate, 1 mM EDTA, 1%Na_3_VO_4_, 0.5 µg/ml leupeptin, 1 mM PMSF. CA916798 complex was immunoprecipitated with anti-CA916798 antibody and detected with anti-phosphorylated AKT1 (ser473) antibody. The P-AKT1 (ser473) complex was immunoprecipitated with anti-phosphorylated AKT1 (ser473) antibody and detected with anti-CA916798 antibody. Protein A-Sepharose beads were added to bind the complex from solution. The complex was brought down in the pellet by centrifugation and boiled in the presence of SDS to liberate antigen. The rest procedures of immuno-blotting were described as mentioned above. Naive IgG was used as negative control.

### Orthotopic Nude Mice Pulmonary Adenocarcinoma Model

Male athymic nude mice (BALB/c *nu/nu*, 4–6 weeks old) were used. A549 or A549/CDDP cells (6×10^5^) in 0.2 ml culture medium were injected subcutaneously into the flank and groin of each nude mouse. Once the subcutaneous tumors reached 0.5 cm in diameter, cisplatin, LY294002 or rapamycin were injected intraperitoneally. Thirty mice were divided randomly into 6 groups according to different administration: (a) A549; (b) A549+ LY294002; (c) A549+ LY294002+ rapamycin; (d) A549/CDDP; (e) A549/CDDP+LY294002; (f) A549/CDDP+LY294002+ rapamycin. LY294002 at 25 mg/kg or rapamycin at 8 mg/kg were given to corresponding groups twice a week for 4 weeks continually. All animals were received 4 mg/kg of cisplatin once a week for 4 weeks continually. Volumes of tumors were calculated (volume = long axis×short axis^2^) once a week. Then mice were sacrificed, tumors were removed and embedded in paraffin. Five µm sections were cut, fixed onto glass slides, and then routine HE stain and immunohistochemical were performed.

### Collection of Human Lung Cancer Specimens

Thirty-eight human adenocarcinoma specimens embedded in paraffin were kindly offered by the Department of Pathology in Southwest Hospital. The specimens were collected from the patients who accepted radical surgery in Southwest Hospital of the Third Military Medical University from January 2008 to November 2011. The pathologic types of lung cancer were determined by the Department of Pathology of Southwest Hospital. We followed up each case for the prognosis. The recurrence or distant transfer of cancer after 3–5 courses of chemotherapy was regarded as the characteristics of drug resistance. The specimens were cut into 5 µm sections, fixed on glass slides. HE stain and immunohistochemical were performed.

### Immunohistochemistry

After antigen retrieval by microwave, sections were stained for the expression of phospho-AKT (1∶50), CA916798 (1∶400) or phospho-mTOR (1∶100), and detected by streptavidinbiotin-horseradish peroxidase complex formation. Antibody against phospho-mTOR (Ser2448) or phospho-AKT (ser473) was purchased from Cell Signaling Technology, and rabbit anti-human CA916798 polyclone antibody was made and gently offered by Dr Haijing Wang [8]. Tumor sections stained without primary antibodies were used as negative control. Quantification for phospho-AKT CA916798 and phospho-mTOR positive cells was done in five arbitrarily selected fields in each tumor sample. Data are represented as the number of positive (brown) cells×100/total number of cells as described before [14].

### Statistical Analysis

All data values in the present study were reported as mean±SD. Student’s unpaired *t* test was used for analyzing the two pairs of data in the experiments. ANOVA was used for comparing the data with more than two treatments in the experiments. Differences between data values were considered statistically significant if P<0.05. All analysis was carried by SPSS16.0.

## Results and Discussions

### Correlation of PI3K/AKT1/mTOR Pathway with CA916798 is Associated with Multi-drug Resistance of Pulmonary Adenocarcinoma

CA916798 is a novel gene related to multi-drug resistance of lung cancer [8], [15]. CA916798 locates in 19q13.33, and contains 5 exons with coding region in the fifth exon. CA916798 gene was identified from cisplatin-resistant human lung adenocarcinoma cell line. The sequence of CA916798 is corresponding to C19orf48, which was identified by the Mammalian Gene Collection Program [16], [17], [18]. Besides its multidrug function, CA916798 carries on other functions in cancer biology. It has been reported that CA916798 encodes a minor histocompatibility antigen recognized by CD8+ cytotoxic T Cells, and may devote to tumor regression in patients with renal cell carcinoma after non-myeloablative allogeneic hematopoietic cell transplantation [19]. CA916798 is also a androgen-responsive gene,which may be target for therapies or biomarkers for prostate cancer [20]. However, it remains unknown about how CA916798 leads to the resistance of chemotherapy.

Many researches reveal that the activation of PI3K/AKT signaling pathway can enhance multi-drug resistance. This signaling pathway is activated in lung cancer cells. Inhibition of PI3K/AKT signaling pathway can dramatically inhibit drug resistance of cancer cell, and induce apoptosis in lung cancer cells [21], [22], [23]. As an important phosphokinase in vivo, AKT is the central member of PI3K/AKT signaling pathway [12], [24]. Three isoforms of AKT, AKT1, AKT2, and AKT3, have been found until now [25]. Previous research showed that A549/CDDP cell line had higher expression level of AKT1, not AKT2 and AKT3, than parent A549 cell line. AKT1 amplification can lead to multi-drug resistance formation in normal pulmonary adenocarcinoma A549 cell line, while inhibition of AKT1 abolishes drug resistance of A549/CDDP cell line, indicating that AKT1 is essential for multi drug resistance [26]. Thus our attentions are focused on the relationship between CA916798 and AKT1 in the following researches.

**Figure 1:**
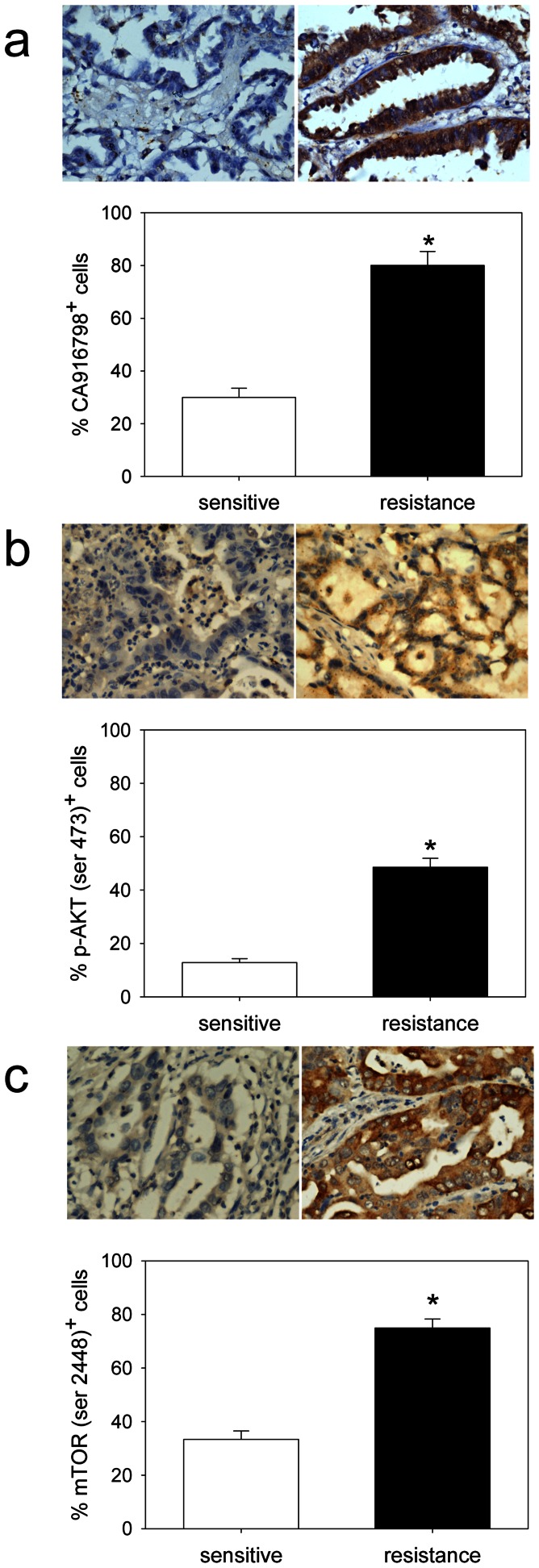
Expression of CA916798, phosphorylated AKT or phosphorylated mTOR in human pulmonary adenocarcinoma tissue as detected by immunohistochemisty images (×400 magnification). a. CA916798 expression (top: a representative image; bottom: semi-quantitative analysis of CA916798 positive cells). *p<0.05: drug resistance *vs.* drug sensitive. b. phosphorylated AKT (top: a representative image; bottom: semi-quantitative analysis of phosphorylated AKT positive cells). c. phosphorylated mTOR (top: a representative image; bottom: semi-quantitative analysis of phosphorylated mTOR positive cells). Data are presented as the mean±SD (sensitive group: n = 18; resistance group: n = 20).

We observed parallel increase of CA916798 protein level with phosphorylation of AKT1. Expression of CA916798 was higher in A549/CDDP cell line than parent A549 cell line. CA916798 amplification enhanced drug resistance of small cell lung cancer cell line H446 that was used to be sensitive to chemotherapy, while inhibition of CA916798 abolished drug resistance of A549/CDDP cell line. In human adenocarcinoma samples, phosphorylated AKT, phosphorylated mTOR, and CA916798 expression level were found all increased in the drug resistance group in parallel with the change of drug resistance (Figure 1).

**Figure 2:**
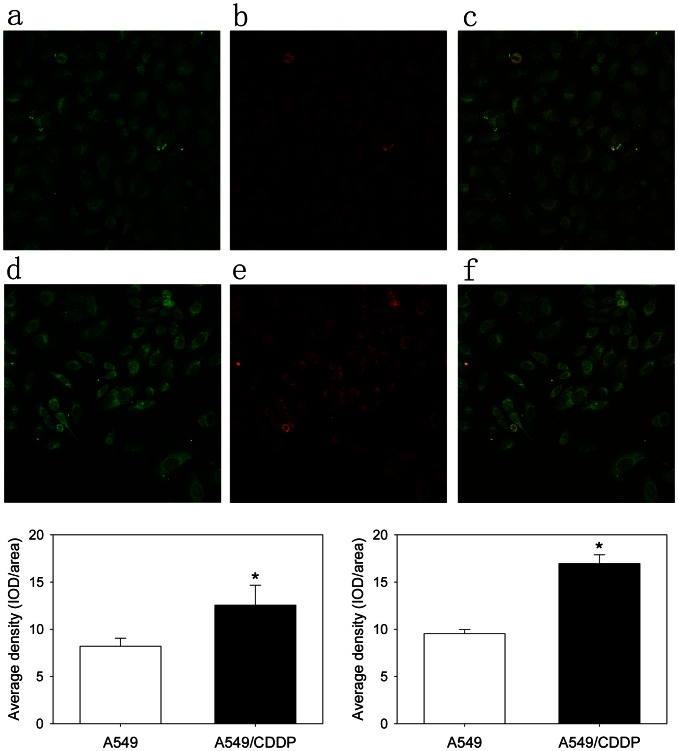
Immunofluorescence staining of CA916798 expression and phosphorylated AKT1 in A549 and A549/CDDP cell lines. a and d: CA916798 (green fluorescence FITC); b and e: phosphorylated AKT1 (red fluorescence CY3); c and f: merged images; a, b, and c: pulmonary adenocarcinoma cell line A549; d, e, and f: multidrug resistance cell line A549/CDDP (Confocal fluorescence microscopy,×400 magnification). *Bottom*: left panel, Levels of Phosphorylated AKT1; right panel, Levels of CA916798 (target protein level were analysis by IPP5.0 program). *P<0.05: A549/CDDP group *vs.* A549 group. Data are presented as the mean±SD (n = 5).

**Figure 3:**
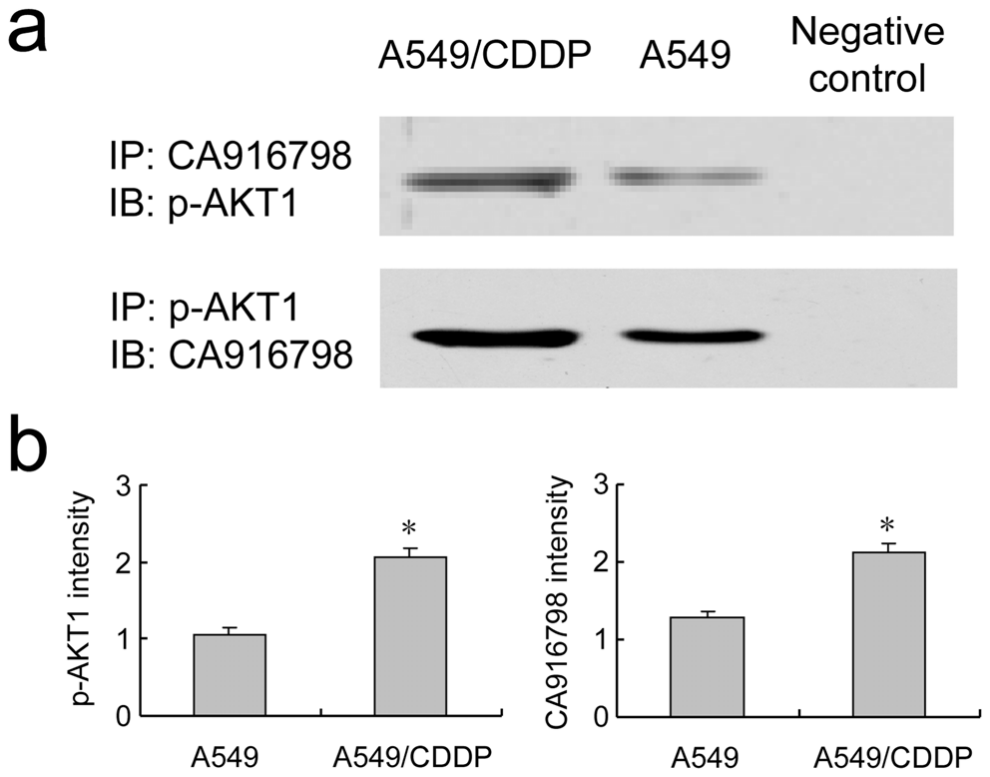
Interaction of CA916798 and p-AKT1 in A549 and A549/CDDP cell lines. Co-immunoprecipitation was employed to detect the interaction of CA916798 and p-AKT1 in A549 and A549/CDDP cell lines. a, IP: CA916798 antibody, and IB: p-AKT1 antibody; IP: p-AKT1 antibody, and IB: CA916798 antibody. b, Semi-quantitative analysis. *P<0.05: A549/CDDP group *vs.* A549 group. Data are presented as the mean±SD (n = 5).

Taking abovementioned results into consideration, we presumed that CA916798 might be related to PI3K/AKT signaling pathway and their relationship might be related to multi-drug resistance. Indeed, as shown in Figure 2 and 3, immunofluorescence and Co-IP showed that: (i) CA916798 located both in the cytoplasm and nuclei of A549 and A549/CDDP cell lines; (ii) higher levels of CA916798 and p-AKT1 could be detected in A549/CDDP cell line than its parental A549 cell line, which was consistent with previous reports; (iii) CA916798 expression was associated with phosphorylation of AKT1 in both A549/CDDP cell line and ordinary A549 cell line, which was much stronger in A549/CDDP cell line than A549 (Figure 2 and 3). The results confirm that CA916798 and p-AKT1 are correlated with each other. Their stronger association in multidrug resistance cell line may be correlated with drug resistance of lung adenocarcinoma.

### Phosphorylation of AKT1 Enhances the Levels of CA916798 mRNA and Protein

**Figure 4:**
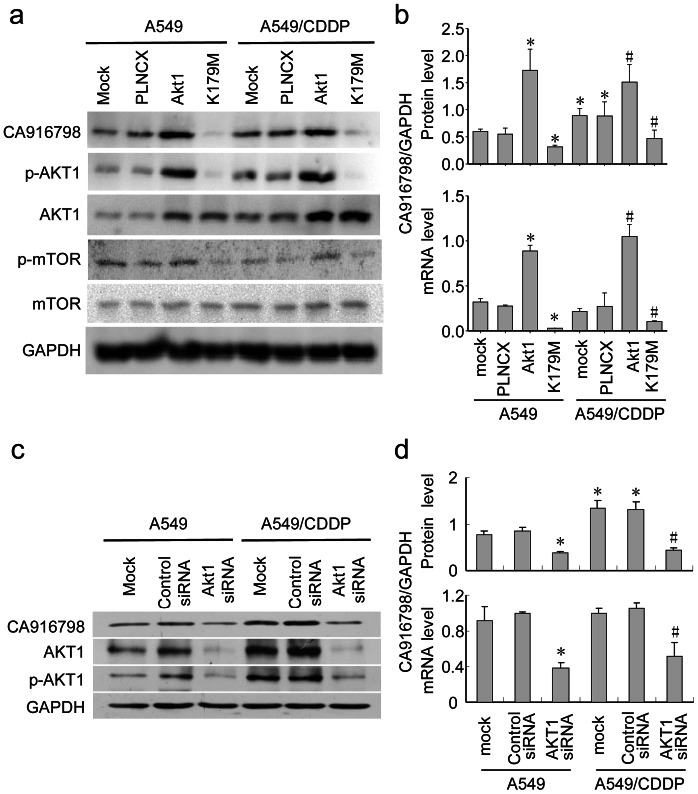
AKT1 regulates CA916798 expression. a, Western blot analysis of CA916798 protein abundance affected by plncx-AKT1-induced AKT1 overexpression or plncx-AKT1-K179M-mediated reduction of AKT1 phosphorylation. GAPDH was used as loading control; b, Quantitative analysis of CA916798 protein abundance (*top panel*) and quantitative real-time PCR (qRT-PCR) analysis of CA916798 mRNA (*lower panel*); c, Western blot analysis of CA916798 protein abundance reduced by siRNA-mediated inhibition of AKT1. GAPDH was used as loading control; d, Quantitative analysis of CA916798 protein abundance (*top panel*) and qRT-PCR analysis of CA916798 mRNA (*bottom panel*). *P<0.05 *vs.* A549; ^#^P<0.05 *vs.* A549/CDDP. Data are presented as the mean±SD (n = 5).

In order to find out the relationship between CA916798 and AKT1, plncx-AKT1 and plncx-AKT1-K179M plasmids were used to transfect A549 or A549/CDDP cell lines to augment or inhibit the level of phospho-AKT1 by the method as described previously [13]. After phosphorylation by PDK1 and mTORC2, AKT becomes activated as phospho-AKT (p-AKT) to activate downstream targets in the nucleus and cytoplasm [27]. As shown in Figure 4, we observed that expression of CA916798 on protein level was correlated with the level of phosphorylated-AKT1; amplification of phosphorylated-AKT1 led by AKT1 plasmid transfection caused higher expression of CA916798 at the levels of protein and mRNA, while inhibition of p-AKT1 by AKT1-K179M plasmid infection down-regulated CA916798 at the levels of protein and mRNA dramatically as well. AKT1 siRNA was also used to knock down AKT1 expression as well as phosphorylated-AKT1. As shown in Figure 4, AKT1 siRNA reduced CA916798 expression on the levels of mRNA and protein dramatically (P<0.05), confirming that CA916798 was the downstream target of AKT1.

### Inhibition of mTOR by Rapamycin or PI3K by LY294002 Reduces CA916798 Expression

**Figure 5:**
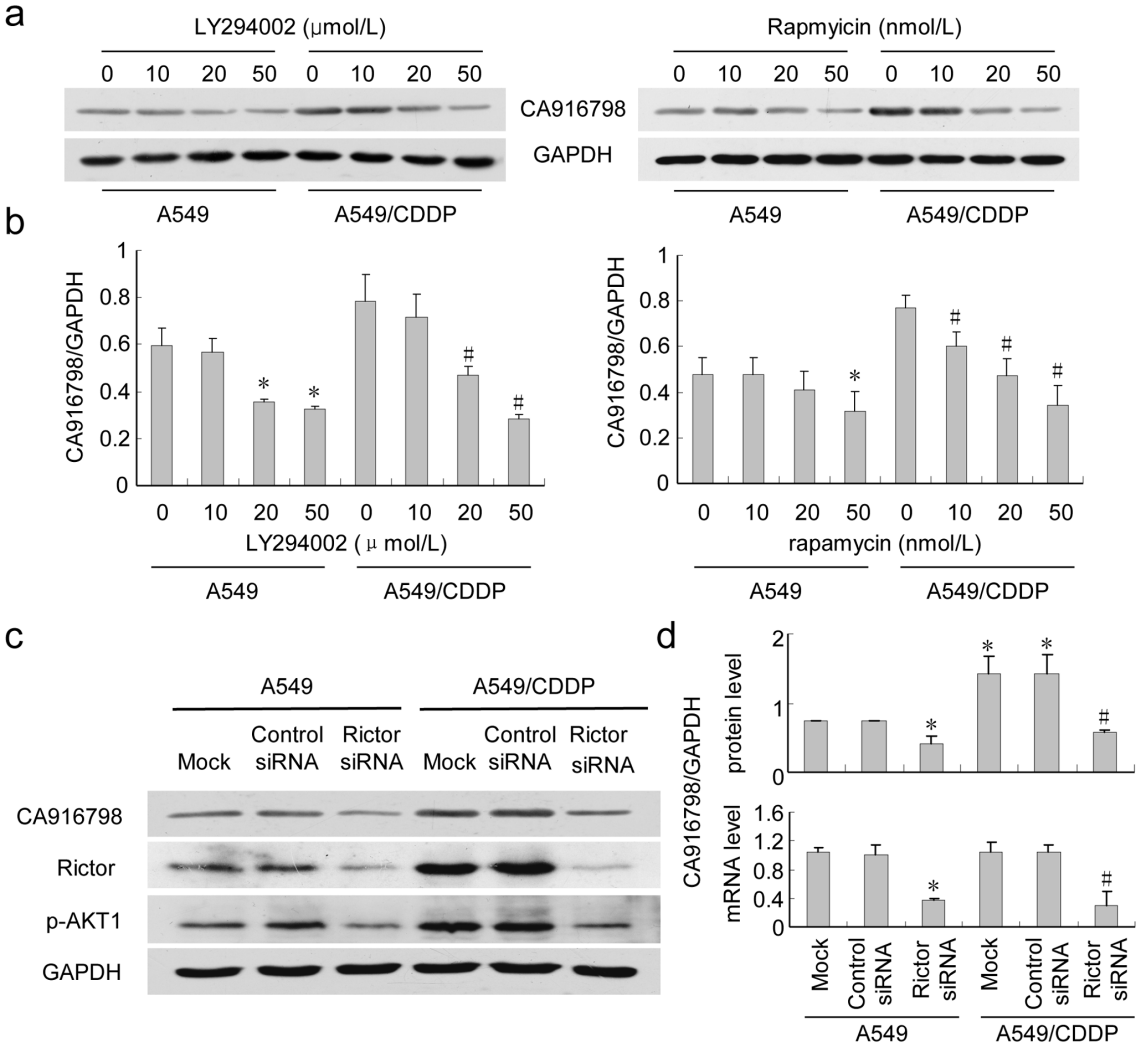
Inhibitions of PI3K or mTORC1/2 reduce CA916798 expression. a, LY294002 and rapamycin inhibit CA916798 expression in a dose-dependent manner. A549 and A549/CDDP cells were treated with LY294002 (0, 10, 20, and 50 µmol/L) (*left panel*) or rapamycin (0, 10, 20, and 50 nmol/L) (*right panel*) for 24 h, Total cellular proteins were extracted and analyzed by western-blot, GAPDH was used as loading control; b, Quantitative analysis of CA916798 protein abundance affected by LY294002 (*left panel*) or rapamicin (*right panel*); c, Rictor inhibition by siRNA reduced CA916798 expression. Total cellular proteins were extracted and analyzed by western-blot, GAPDH was used as loading control; d, Quantitative analysis of CA916798 protein abundance affected by siRNA-mediated Rictor inhibition (*top panel*), and qRT-PCR analysis of CA916798 mRNA level (*bottom panel*).*P<0.05 *vs.* A549; ^#^P<0.05 *vs.* A549/CDDP. Data are presented as the mean±SD (n = 3).

LY294002 is a well-known inhibitor of PI3K, which inhibits activation of AKT. As shown in Figure 5, LY294002 administration repressed CA916798 expression on he levels of mRNA and protein dramatically (P<0.05), confirming that CA916798 was the downstream target of PI3K/AKT pathway (Figure 5, Figure S1 and Figure S2).

MTOR is an important downstream phosphorylase of PI3K/AKT signaling pathway. AKT activates mTOR to become phosphorylated mTOR (p-mTOR) through TSC1/2. Phospho-mTOR has two forms with active phosphorylation capability as mentioned above: mTORC2 phosphorylates and actives AKT [28], while mTORC1 phosphorylates and activates downstream targets eLF or S6K to increase cell growth, survival, and proliferation [29], [30]. As an mTORC1 inhibitor, rapamycin or its analogs can block the corresponding functions of mTORC1 to enhance the sensitivity of tumor cells to chemotherapy [29], [31], [32].

As shown in Figure 4, CA916798 expression not only paralleled with AKT1 phosphorylation, but also phosphorylation of mTOR (P-mTOR). It has been reported that AKT1 regulate multi-drug resistance through mTOR [26]. Thus we presumed that mTOR might participate in the regulation of CA916798 by AKT1. As shown in Figure 5, administration of rapamycin led to the inhibition of mTORC1 and suppressed CA916798 either in normal A549 cell line or in A549/CDDP cell line (Figure 5, and Figure S1 and S2), indicating that AKT1 regulates CA916798 expression through mTOR-dependent pathway.

To validate the role of mTORC2 in CA916798 expression, Rictor siRNA was used. As shown in Figure 5, Rictor siRNA administration repressed CA916798 at the levels of mRNA and protein dramatically (P<0.05), confirming that CA916798 is the downstream target of mTORC2 as well. mTORC2 may be through EGFRvIII-mTORC2 -NFκB pathway to participate in the cisplatin resistance of glioblastoma [33]. Our research may indicate a novel mechanism for mTORC2 in the chemotherapy resistance of tumor.

It has been reported that PI3K/AKT/mTOR signaling pathway may regulate the expressions of other genes through direct regulation of corresponding transcription factors, such as SP-1 [34], suggesting that PI3K/AKT1/mTOR may regulate expression of CA916798 through regulating interaction of some transcription factors with the promoter of CA916798 gene.

### Inhibition of CA916798 has no Effect on the Expression of AKT or mTOR

**Figure 6:**
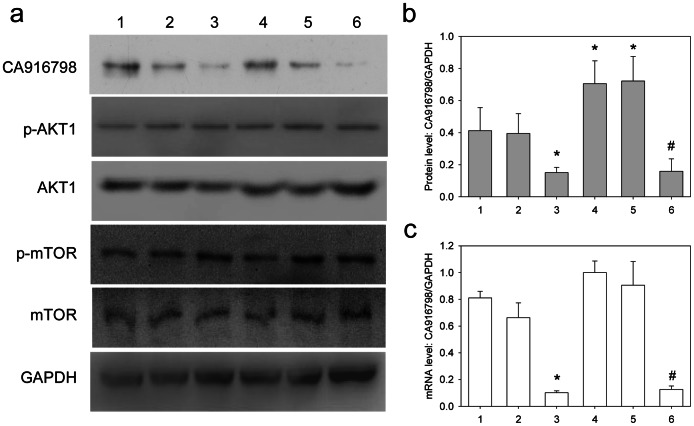
Inhibition of CA916798 has no effect on the levels of mRNA and protein as well as phosphorylated AKT1 or mTOR. a, CA916798, phosphorylated AKT1, AKT1 protein, phosphorylated mTOR, and mTOR protein were determined by Western blot analysis. GAPDH was used as loading control; b, Relative protein level of CA916798; c, relative mRNA level of CA916798. Lane 1, A549; Lane 2, A549+ PGCSI empty vector; Lane 3, A549+PGCSI-CA916798-ShRNA; Lane 4, A549/CDDP; Lane 5, A549/CDDP+PGCSI empty vector; Lane 6, A549/CDDP+ PGCSI-CA916798-ShRNA. *P<0.05 *vs.* A549; ^#^P<0.05 *vs.* A549/CDDP. Relative expression level of AKT1 and mTOR was not changed in mRNA, total protein or phosphorylated protein level (data not showed).

In order to address whether CA916798 expression had any effect on PI3K/AKT pathway, we constructed shRNA against CA916798 and found that it reduced CA916798 expression efficiently (Figure 6). However, such shRNA-mediated decrease of CA916798 did not cause any changes of PI3K/AKT1/mTOR signaling pathway in either A549 or A549/CDDP cell lines (Figure 6). This result further strengthens our conclusion that CA916798 is the downstream target of PI3K/AKT1/mTOR pathway.

### Orthotopic Nude Mouse Pulmonary Adenocarcinoma Experiments Confirm that CA916798 is the Downstream Target of PI3K/AKT1/mTOR Signaling Pathway

**Figure 7:**
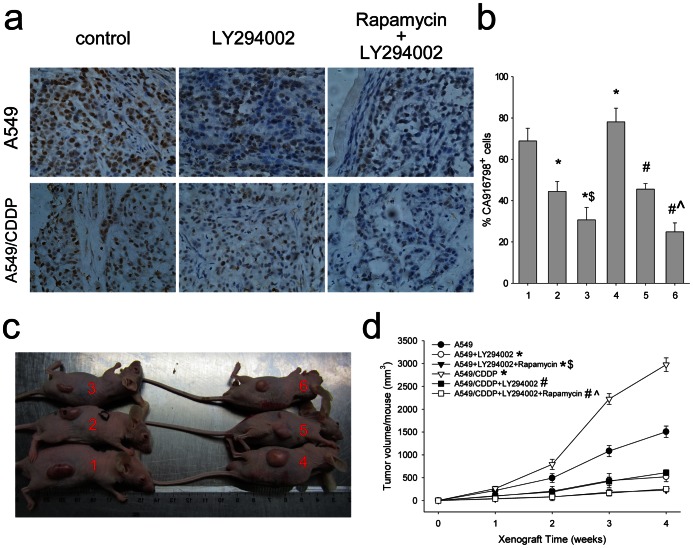
Inhibition of Phosphorylated AKT1 or Phosphorylated mTOR reduces CA916798 expression in pulmonary adenocarcinoma xenografts. a, Immunohistochemisty images (×400 magnification); b, Quantitative analysis of corresponding positive cells; c, Representative tumor-possessing nude mice; d, Average tumor volume of the corresponding week. 1, A549; 2, A549+LY294002; 3, A549+LY294002+rapamycin; 4, A549/CDDP; 5, A549/CDDP+LY294002; 4, A549/CDDP+LY294002+rapamycin. *: p<0.05 *vs.* A549; #: p<0.05 *vs.* A549/CDDP; $: p<0.05 *vs.* A549+LY294002; ^∧^: p<0.05 *vs.* A549/CDDP+LY294002. The comparisons were between tumor volumes of the fourth week. Data are presented as the mean±SD (n = 5).

In order to investigate the role of CA916798 on the drug resistance in vivo, all mice were received cisplatin during the experiment process. In the process of experiment, all groups of nude mice behaved normally for growth, diet and activity; and obvious reduction of body weight was not observed. As shown in Figure 7, appearances of tumor were spherical large tumor nodule or several small tumor nodules fasciculation. For tumor size, LY294002 group was smaller than control group, while tumor size of A549/CDDP group are bigger than A549 group. The levels of p-AKT and p-mTOR were strongly inhibited by LY294002 both in A549 and A549/CDDP tumor xenografts, indicating that this drug blocked AKT/mTOR pathway efficiently.

In the A549 cells cultured in vitro, we found that LY294002 or rapamycin strongly depressed CA916798 expression, suggesting that CA916798 may be the downstream factor of AKT/mTOR pathway. We performed microscopic analysis on the A549 tumor xenografts and found that CA916798 immunostaining intensity was reduced by 24% after LY294002 treatment as compared with controls (Figure 7a and b). Additionally, microscopic analysis of CA916798 immunostaining intensity in the A549/CDDP tumor xenografts showed 33% reduction for LY294002-treated mice as compared with controls (Figure 7a and b). Thus, LY294002 can reduce CA916798 expression efficiently in the xenograft models, confirming that CA916798 is the downstream factor of PI3K/AKT/mTOR pathway in vivo.

The combination effect of rapamycin and LY294002 on the expression of CA916798 in vivo was also investigated. As shown in Figure 7a and b, rapamycin and LY294002 effectively reduced CA916798 positive cells in the A549 tumor xenografts by 31% reduction as compared with LY294002 treated solely. Tumor size of LY294002 plus rapamycin treated group was smaller than that of LY294002 group (Figure 7c and d). In A549/CDDP tumor xenografts, CA916798 immunostaining intensity showed 45% reduction for the mice treated with LY294002 plus rapamycin as compared with LY294002 group (Figure 7a and b). Tumor size of LY294002 plus rapamycin treated group was smaller than that of LY294002 group significantly (Figure 7c and d). Of note, all mice remained survival, and the survival rate are 100% after intraperitoneal injection of the abovementioned compounds for 4 weeks (Data not shown). Thus, LY294002 plus rapamycin exerts synergistic effects on the reduction of CA916798 expression along with tumor growth in either A549 or A549/CDDP xenograft models.

Similar structural homology of catalytic domains is shared by p110 subunits and mTORC1. Therefore, inhibitors of PI3K and mTORC1 may have synergistic effects theoretically. BeZ235 is an inhibitor of PI3K and mTOR, and its inhibitory effect on the growth of NSCLC cells and tumor xenografts can be strengthened by mTORC1 inhibitor everolimus [9], [35]. The synergistic effect was interpreted by the uncouple feedback inhibition between S6K and PI3K [9]. However, our data indicate that this synergistic effect may be caused through CA916798. Nevertheless, we still need additional studies to explore the underlying mechanisms in more detail.

In summary, we observed that the levels of phosphorylated AKT, phosphorylated mTOR and CA916798 all increased in the specimens excised from the patients with drug resistant human adenocarcinoma and paralleled with the change of drug resistance. We further demonstrated that inhibition of AKT1, mTORC1, mTORC2 and PI3K reduced CA916798 expression, while overexpression of AKT1 augmented CA916798 expression. Inhibition of PI3K by LY294002 could efficiently reduce CA916798 expression and tumor size in a xenografted tumor model as well. Additionally, the inhibitory effects of LY294002 combined with rapamycin on CA916798 expression and tumor size were stronger than LY294002 alone. Our findings identify that CA916798 is the downstream target of PI3K/AKT/mTOR pathway, which will help us to understand the underlying mechanisms how CA916798 participate in multi-drug resistance of cancer.

## Supporting Information

Figure S1 **Both LY294002 and rapamycin can inhibit the proliferation of A549 and A549/CDDP cell lines in the dose-dependent manner.** There were no significant difference between the cell proliferation of A549 and A549/CDDP cell lines upon treated by LY294002 or rapamycin for 48 hours (mean±SD, n = 5).
(TIF)

Figure S2 **LY294002 plus rapamycin inhibit cell viability of A549 and A549/CDDP cells more significantly than LY294002 or rapamycin alone.** Proliferation of cells without LY294002 and rapamycin (medium) were set as 100%; ly: LY294002 (50 µmol/l); rap: rapamycin (50 nmol/l); *p<0.05 vs. A549; #p<0.05 vs. A549/CDDP; △p<0.05 vs. A549+LY294002; ▴p<0.05 vs. A549+rapamycin; a p<0.05 vs. A549/CDDP+LY294002; b p<0.05 vs. A549/CDDP +rapamycin (mean±SD, n = 5). The drugs were administrated for 48 hours.
(TIF)

Table S1 **IC50 and drug resistance of A549/CDDP to different chemotherapeutics.**
(DOC)
