# Supplementary figures and images for: Akt1 Enhances CA916798 Expression through mTOR Pathway
Source: PLoS One. 2013 May 8;8(5):e62327. doi: 10.1371/journal.pone.0062327 (PMC3648559; doi:10.1371/journal.pone.0062327)

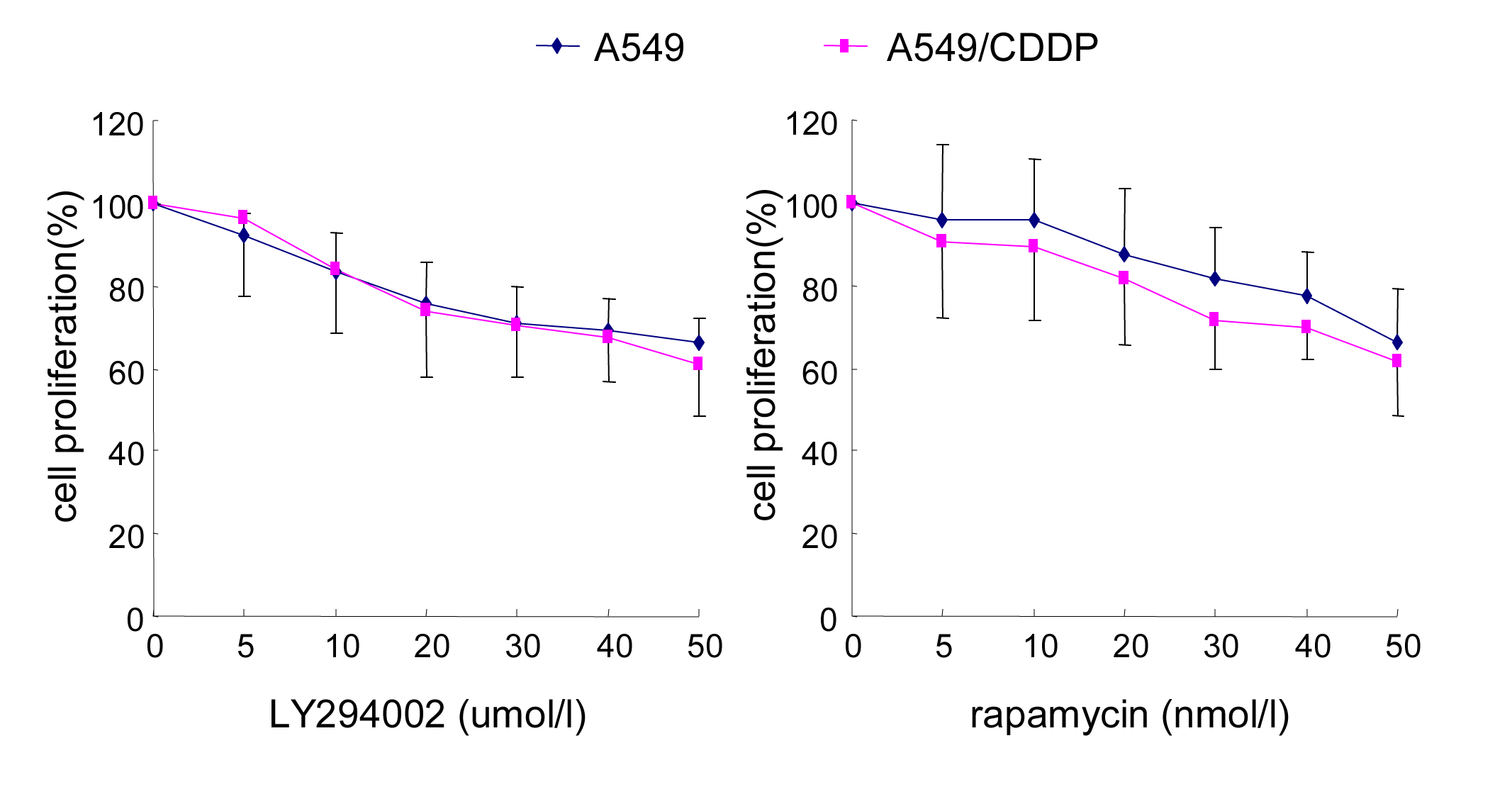

Supplement: Figure S1 — Both LY294002 and rapamycin can inhibit the proliferation of A549 and A549/CDDP cell lines in the dose-dependent manner. There were no significant difference between the cell proliferation of A549 and A549/CDDP cell lines upon treated by LY294002 or rapamycin for 48 hours (mean±SD, n = 5). (TIF) [file pone.0062327.s001.tif]

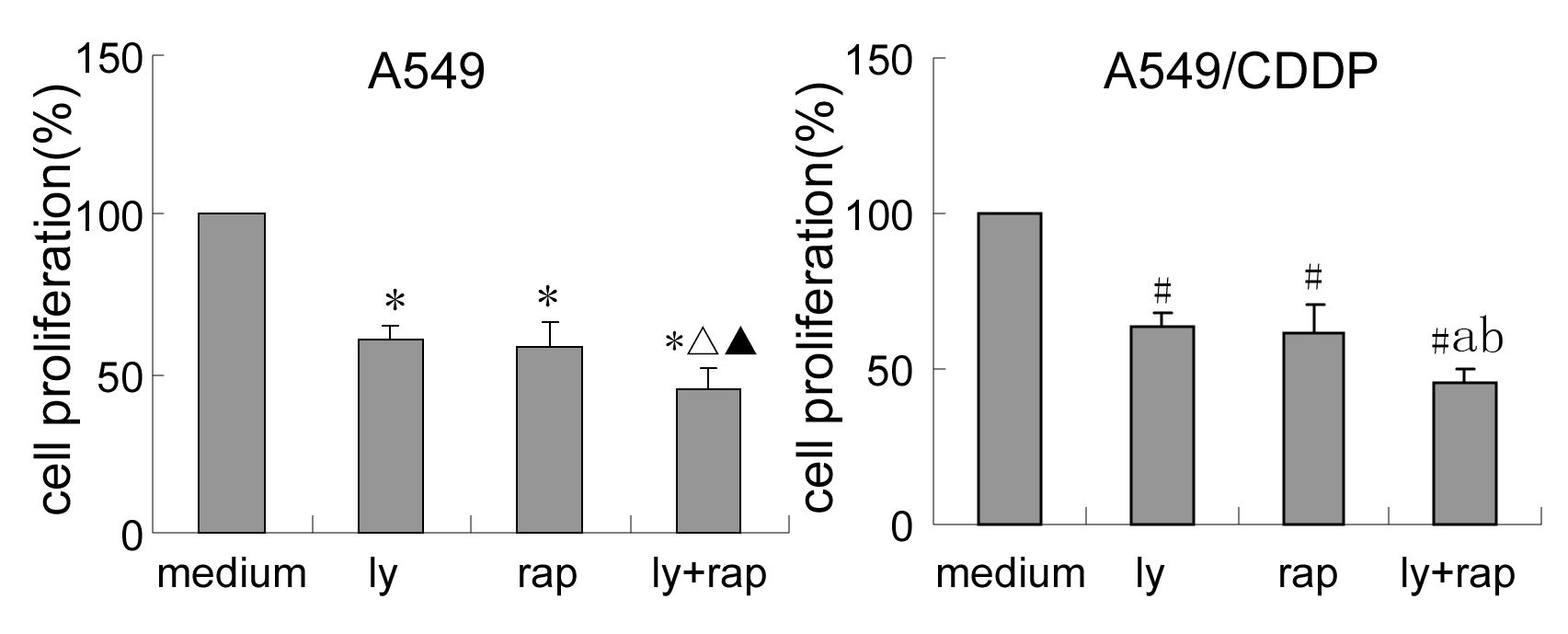

Supplement: Figure S2 — LY294002 plus rapamycin inhibit cell viability of A549 and A549/CDDP cells more significantly than LY294002 or rapamycin alone. Proliferation of cells without LY294002 and rapamycin (medium) were set as 100%; ly: LY294002 (50 µmol/l); rap: rapamycin (50 nmol/l); *p<0.05 vs. A549; #p<0.05 vs. A549/CDDP; △p<0.05 vs. A549+LY294002; ▴p<0.05 vs. A549+rapamycin; a p<0.05 vs. A549/CDDP+LY294002; b p<0.05 vs. A549/CDDP +rapamycin (mean±SD, n = 5). The drugs were administrated for 48 hours. (TIF) [file pone.0062327.s002.tif]
