# Supplementary material for: Akt1 Enhances CA916798 Expression through mTOR Pathway
Source: PLoS One. 2013 May 8;8(5):e62327. doi: 10.1371/journal.pone.0062327 (PMC3648559; doi:10.1371/journal.pone.0062327)
Supplement: Table S1 — IC50 and drug resistance of A549/CDDP to different chemotherapeutics. (DOC) [file pone.0062327.s003.doc]

**1. Identification of A549/CDDP multi-drug resistance**

We chose seven different chemotherapeutics to administrate A549 and A549/CDDP cells for 48 hours. Each chemotherapeutics set 5 different concentrations and 5-fold difference between each concentration. MTT assay was used to generate IC50.

Cells were seeded into 96-well plates for 5×10^3^ per well overnight, after cells were adherent normally, different drugs in different concentrations were added. After culture for 48 h, MTT dye solution was added to each well and samples were incubated at 37℃ for 4 h. The formazan product was dissolved by adding 200µL of DMSO to each well and the plates were read at 570 nm. The IC50 value was calculated from the 50% formazan formation compared with a control without the addition of chemotherapeutics. All measurements were done in triplicate wells.

Table S1. IC50 and drug resistance of A549/CDDP to different chemotherapeutics

| chemotherapeutics | IC50 (μg/ml) (mean±SD, n=3) | | Drug resistance |
| --- | --- | --- | --- |
|  | A549 | A549/CDDP |  |
| cisplatin | 1.912±0.63728 | 15.413±2.9265** | 8.041127 |
| paclitaxel | 0.426±0.216736 | 4.239±0.7481613** | 9.951487 |
| pemetrexed | 1.188±0.015144 | 1.81±0.31031* | 1.522715 |
| epirubicin | 0.175±0.005568 | 0.655±0.283988** | 3.746667 |
| mitomycin | 0.765±0.121858 | 1.161±0.145634* | 1.517422 |
| gemcitabine | 0.103±0.028931 | 0.628±0.082397** | 6.10356 |
| carboplatin | 0.358±0.070117 | 1.813±0.231461** | 5.061395 |

* p<0.05 vs. A549 ** p<0.01 vs. A549
